# Nonlinear thinking in ecology and evolution: applying the threshold elemental ratio across levels of ecological organization

**DOI:** 10.1007/s00442-025-05842-w

**Published:** 2025-12-08

**Authors:** Benjamin B. Tumolo, Carly R. Olson, Erin I. Larson, Halvor M. Halvorson, Catherine E. Wagner, Amy C. Krist, Felicia S. Osburn, Eric K. Moody, Linnea A. Rock, Uchechukwu V. C. Ogbenna, Eli N. Wess, Briante Najev, Anthony J. Pignatelli, Jessica R. Corman

**Affiliations:** 1 Department of Zoology and Physiology, Program in Ecology and Evolution, University of Wyoming, Laramie, WY USA; 2 Department of Biology, Northern Michigan University, Marquette, MI USA; 3 School of Natural Resources, University of Nebraska-Lincoln, Lincoln, NE USA; 4 Wyoming Cooperative Fish & Wildlife Research Unit, University of Wyoming, Laramie, WY USA; 5 Alaska Center for Conservation Science, University of Alaska Anchorage, Anchorage, AK USA; 6 Department of Biology, University of Central Arkansas, Conway, AR USA; 7 Department of Botany, Program in Ecology and Evolution, University of Wyoming, Laramie, WY USA; 8 Department of Biology, Middlebury College, Middlebury, VT USA; 9 Department of Biology, University of Iowa, Iowa City, IA USA; 10 Department of Biological Sciences, University of South Carolina, Columbia, SC USA

**Keywords:** Biogeochemistry, Ecological stoichiometry, Ecological threshold, Evolutionary threshold, Spatiotemporal scale

## Abstract

Nonlinear dynamics govern ecological processes; thus, understanding thresholds is important for measuring and forecasting the effects of climate change and management of natural resources. However, identifying whether and how such thresholds transfer across ecological levels of organization remains challenging. We argue for a broadening of a foundational organismal concept from ecological stoichiometry theory, the threshold elemental ratio (TER), to study how nonlinear dynamics driven by shifts in limitation operate in evolutionary and ecological processes from organisms to ecosystems. Traditionally, TERs are used to describe the elemental ratio at which the limitation of organismal growth shifts from one element to another. Building on this definition, we make a case for broadening the TER beyond organisms to include populations, clades, communities, and ecosystems. We discuss how TERs may be detected and translated across different ecological levels and evolutionary processes through simulation modeling, literature review, and synthesis of empirical examples from diverse systems and scales including: cyanotoxin production in lakes, alder–salmon dynamics, and the Cambrian explosion. Collectively, we argue that TERs are likely widespread and consequential across levels of ecological organization and that such thresholds manifest from a diversity of mechanisms. Thus, applying the TER concept across ecological levels of organization holds promise for advancing our understanding of nonlinear dynamics from the micro-evolutionary to the macro-ecological.

**Supplementary Information:**

The online version contains supplementary material available at 10.1007/s00442-025-05842-w.

## Introduction

Thresholds in ecological and evolutionary relationships alter the trajectories of populations, communities, ecosystem processes, and evolutionary rates. Often the existence, location, and timing of thresholds are signaled by nonlinear relationships among ecological variables (Scheffer and Jeppesen 2007; Ratajczak et al. 2018; Spake et al. 2022). Thus, identifying the conditions required for thresholds to occur is foundational to understanding dynamic ecological processes and systems. For example, understanding thresholds is critical to measuring and forecasting the effects of climate change and for management of natural resources (Dodds et al. 2010). Despite significant progress in identifying the causes and consequences of thresholds to ecological and evolutionary processes, the challenge of how to unify these phenomena across ecological levels of organization remains. Ecological stoichiometry—the study of the balance of multiple chemical elements and energy in ecological systems—is an attractive framework for translating nonlinear dynamics and shifts in nutrient limitation across the ecological hierarchy (i.e., organisms to ecosystems). Ecological stoichiometry characterizes interactions between organisms and their environment using a common currency (i.e., chemical elements and energy) grounded in the first principle of conservation of mass and energy (Sterner and Elser 2002). A key concept from ecological stoichiometry, the threshold elemental ratio (hereafter TER, Olsen et al. 1986; Urabe and Watanabe 1992), describes the nonlinear change in organismal growth or fitness when resource limitation switches from one element to another (Sterner and Elser 2002; Frost et al. 2006). The TER concept is rooted in stoichiometric models that couple organismal bioenergetics and body elemental composition (Frost et al. 2006). Yet, despite a few examples where the TER has been considered at the community (e.g., Elser et al. 1998; Moody et al. 2019) and ecosystem levels (e.g., Elser and Urabe 1999; Mooshammer et al. 2014), TERs have predominantly been applied at the organism level. However, because all organisms require carbon (C), nitrogen (N), and phosphorus (P) for growth and metabolism, insufficient supply of one or more of these essential elements is expected to control process rates from biogeochemical transformations to community structure (Urabe and Watanabe 1992; Elser et al. 2007; Schade et al. 2011; Tromboni et al. 2018), as well as rates of microevolutionary and macroevolutionary processes (Elser et al. 2006; Frisch et al. 2014). Despite the potential for shifts in nutrient limitation to result in thresholds across ecological levels, very few works have examined this possibility via ecological stoichiometry theory, or specifically TERs. Given that the TER concept couples nonlinear change and the effects of relative amounts of elements on biological processes, we suggest that broadening the TER concept and definition beyond the organism level will provide testable predictions that could advance our understanding of nonlinear dynamics driven by shifts in nutrient limitation across ecology and evolution more broadly.

In support of broadening the TER concept, we show how stoichiometry can control nonlinear responses across ecological levels of organization. We argue that broadening the TER concept is needed to integrate the fundamental contributions of ecological stoichiometry across evolutionary and ecological processes at multiple levels of organization. To make this case, we begin by providing a background on the application of TERs to date. Then, we discuss the prevalence and importance of TERs from a diversity of ecosystems and ecological and evolutionary processes by compiling and describing case studies from the literature. Next, we present results from a literature review and a simulation model to evaluate the applicability of translating the TER concept to contemporary questions across ecological levels of organization. We conclude by providing recommendations and considerations for broadening the TER concept and by highlighting areas of future research.

## Threshold elemental ratio across the ecological hierarchy

**Fig. 1:**
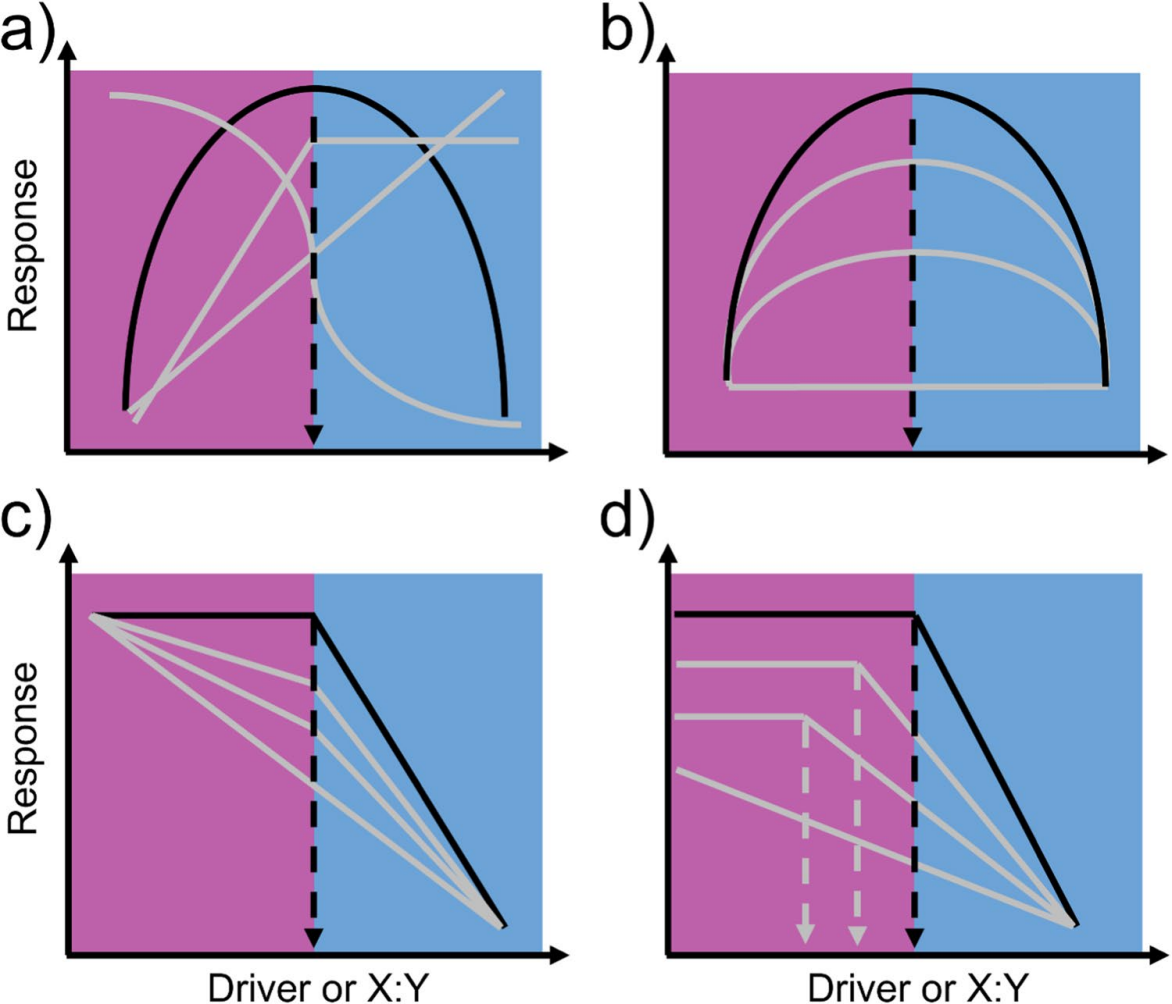
Conceptual visualization of broadening the threshold elemental ratio (TER). TER relationships are black, and theoretical variations from this TER are gray. Shading in panels indicates ranges below (purple; left) or above (blue; right) the focal TER. (**a)** The TER may result from a diversity of potential nonlinear relationships between the stoichiometric driver or X:Y ratios (indicating the ratio of two elements X and Y) and the response. (**b**) TER scenarios may differ in overall sensitivity above and below the TER. (**c**) Across different scenarios, the degree of linearity vs. nonlinearity may also differ. (**d**) The TER itself may differ, depending on the system attributes or environmental drivers

Our proposed broadening of the TER concept is based on the ratio of elements at which an organism, population, community, or ecosystem exhibits a nonlinear change driven by a potential shift in limitation of an ecological or evolutionary state or process (Fig. 1). Our definition of a TER builds on the foundation of the classical definition (Olsen et al. 1986; Urabe and Watanabe 1992) and moves beyond the organism level to be applied to and include population, clade, community, ecosystem, and macrosystem levels. As such, the TER could pertain to a wide range of time scales (physiological to evolutionary) and spatial scales (microscopic to global) (Table 1; Table S1). For example, a TER may reflect a shift in the element most limiting to community-level coexistence among competitors (Tilman 1982) or ecosystem nutrient use efficiency (Mooshammer et al. 2014). Translating the TER across ecological levels of organization may provide a means of explaining nonlinear responses in diverse situations where the currencies of available matter and energy govern the response. Mathematically, the TER constitutes the ratio at which an inflection point, threshold, or break occurs in a relationship between the stoichiometric driver and response variable (Fig. 1a). Thus, identifying and quantifying TERs at various levels of organization can be done thoroughly. To infer that such patterns are being driven by variation in stoichiometry and shifts in nutrient limitation will require robust characterization and consideration of nutrient use efficiency across and at various levels of ecological organization (Hodapp et al. 2019). Nonlinearity is common within ecological dynamics (Dodds et al. 2010; Spake et al. 2022), and many of these relationships, when they involve changes in stoichiometric ratios, may qualify as a TER, but are not captured within the scope of the current framework (Table 1; Table S1). Examining these potential examples with a broader TER concept could improve our understanding of nonlinearity in ecology and evolution in an integrated manner, with shifts in nutrient limitation as the common underlying mechanism.

**Table 1:** Examples of elemental stoichiometry driving nonlinear thresholds at various ecological, evolutionary, spatial, and temporal scales. Grain temporal is the temporal scale of individual observation units; grain spatial is the spatial scale of individual observation units; extent temporal is the temporal scale of the entire study; extent spatial is the spatial scale of the entire study

Topic	Elements	Ecosystem	Driver	Level of Org	Grain		Extent		Response	References
					Temporal	Spatial	Temporal	Spatial		
Adaptive radiation of *Geissois *sp.	Ni, Al, P, Mg, Ca, Co, Cr, Fe	Terrestrial: tropical forest	Ultramafic soil	P: ecosystem (soil) R: community (speciation rate)	NA	Species within a genus	7 m. years	New Caledonia	Threshold	Pillon et al. (2014)
Global leaf decomposition	C, N, P	Aquatic and terrestrial	Vegetation stoich	P: organismal (species C:N:P) R: ecosystem (decomposition rate)	NA	NA	Global	Global	Loglinear	Enriquez et al. (1993)
Streamwater chemistry affects periphyton community	C, N, P	Aquatic: experimental stream	Streamwater stoich	P: Ecosystem (water N:P ratio) R: ecosystem (periphyton N:P, C:N, C:P) and community (chlorophyll)	3 sampling days	Clay tile (0.5 mm^2^)	28 days	30 flumes (2.8 m × 10 cm)	Threshold	Stelzer & Lamberti (2001)
Microbial efficiency	C, N	Terrestrial: soils	Soil N availability	P: Ecosystem (resource, C:N, stoich) R: population (N use efficiency)	one time point	Within same latitude and longitude	5 years	Global	Threshold	Mooshammer et al. (2014)
Invasive fish effect river water N and P	N, P	Aquatic: rivers	Invasive fish excreting at high N:P	P: organismal/population (abundance) R: Ecosystem (N and P availability)	Hrs–days	Grab samples	hrs-yrs	550 m reach	Threshold	Capps and Flecker (2013)
Cambrian explosion	P	Aquatic: Proterozoic—Cambrian oceans	Phosphogenic event	P: ecosystem (C:P) R: organismal (growth rates, metabolism, evolution of skeletons)	NA	NA	20–30 m. years	Global	Threshold	Cook (1992)
Database modeling of CNP influence on soil bacterial diversity	C, N, P	Terrestrial (soil)	Resource organic soil matter C:N:P	P: ecosystem (soil C:N:P ratio) R: community (Shannon diversity)	Single time point	20 × 20 km^2^ sampling grid	4 yrs	6 ecosystem types	Threshold	Delgado-Baquerizo et al. (2017)
Organic matter stoich and stream N, P uptake	N, P	Aquatic: forested stream	Organic matter N:P	P: ecosystem (organic matter stoichiometry) R: Ecosystem (N:P uptake)	Single time point	10–20 m	1 yr	160 m	Threshold	Gibson and O'Reilly (2012)
Soil microbial carbon use efficiency	C, N	Aquatic: soils	Organic matter N	P: ecosystem (C:N nutrient availability) R: ecosystem (carbon use efficiency)	NA	NA	NA	Global	Threshold	Manzoni et al. (2012)
Eutrophication destabilizes ecosystem	N, P	Aquatic: lakes	Increased P availability	P: Ecosystem (amount of nutrients in the sediment) R: community (shift in macrophyte)	Single time point	0.2 m^2^	2 years	46 lakes in the Yangtze River basin, China	Threshold	Su et al (2019)
Ecosystem N processing rates	C, N	Ocean, great lakes, and streams	Increased resource organic carbon	P: ecosystem (organic carbon:nitrate ratio in water) R: community (microbial production)	NA	NA	Multiple years (data from multiple databases)	Global	Threshold	Taylor and Townsend (2010)

*Level of Org* level of ecological organization (organismal, community, ecosystem), *P* predictor, *R* response, *stoich* stoichiometry

**Fig. 2:**
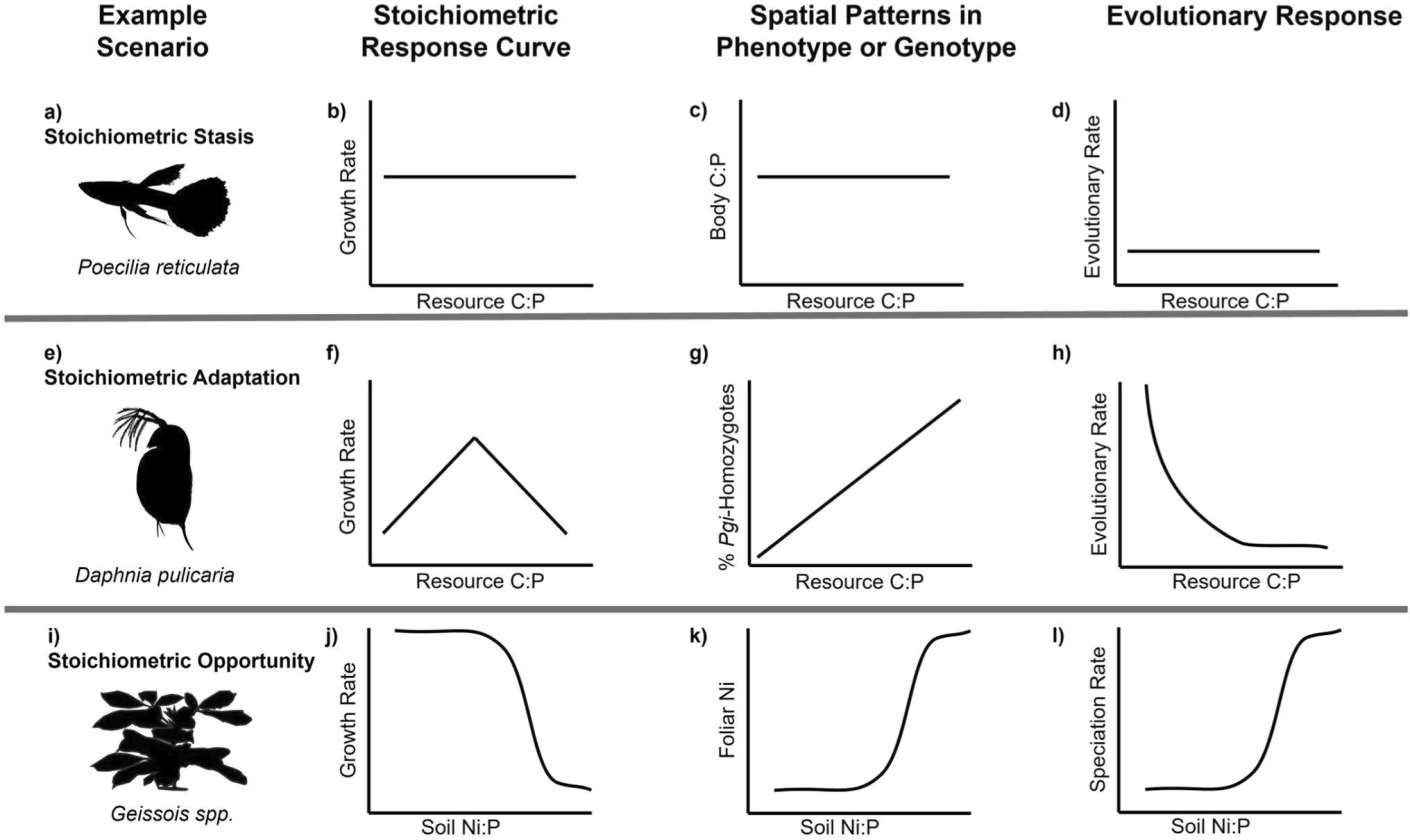
Example response curves for resource stoichiometry and the adaptive responses that might result from them. Plots depict fitness response curves of individual organisms, patterns in phenotype or genotype among populations at a given time, and patterns in evolutionary rate for a population as a function of stoichiometric drivers. (**a**–**d)** Fitness, phenotypic, genotypic, and evolutionary rate response curves are all invariant with resource stoichiometry; there is no threshold on evolutionary processes (El-Sabaawi et al. 2012). (**e**–**f)** Fitness exhibits a unimodal or knife-edge relationship with resource stoichiometry (Moody et al. 2022). (**g)** Phenotypic or genotypic responses vary linearly over space, (**h**) but evolutionary rate varies over time; thus, a threshold exists for organismal performance and for evolutionary rate, but is not apparent when sampling over space (Moody et al. 2022). (**i**–**l)** Nonlinear threshold fitness response curves drive nonlinear responses for the phenotype or genotype and for evolutionary rates corresponding with nickel concentration in soil (Pillon et al. 2014). A threshold is evident across temporal and spatial scales

Many factors are expected to estimate the occurrence and characteristics of stoichiometrically driven thresholds at various ecological levels of organization. Some ecological scenarios may exhibit more sensitivity to thresholds driven by stoichiometry compared to others (Fig. 1b). For example, in a synthesis of soil microbial carbon use efficiencies, sensitivity to substrate C:N was lower in soils that supported lower plant growth compared to soils that supported high plant growth, likely because of an overall limitation of maximum carbon use efficiency in the soils that supported less plant growth (Manzoni et al. 2012).Within a single study system, a threshold may occur at one level of the ecological organization, but not at another; this mismatch may reflect differing time frames required for the threshold to take effect, or it may reflect decoupling among variables. As an example, in an experiment using the herbivorous zooplankter *Daphnia magna* fed a wide dietary C:P gradient, *Daphnia* sp. growth rate exhibited a nonlinear response that peaked at intermediate C:P; yet, feeding rates decreased linearly with decreasing diet C:P (Plath and Boersma 2001). Likewise, evolutionary responses may be linear at the scale of genotypic response, but nonlinear at the level of clade-level diversification rate (Fig. 2). In this way, the same driver may elicit a nonlinear response of one variable but a linear effect of another; the nature of the nonlinearity (e.g., exponential, logistic, degree of nonlinearity) may differ between two responses to the same driver (Fig. 1a, c). Finally, the threshold itself may depend on system attributes or environmental drivers (Fig. 1d) as evidenced by shifts of organism TERs in response to temperature (Laspoumaderes et al. 2022). These examples highlight that numerous different nonlinearities can be observed at one resolution, yet it is difficult to scale these to the next level of ecological organization. Thus, there is an argument to be made that shifts in limitation at the community and ecosystem levels may be an emergent property. Ultimately, parsing the nature and determinants of stoichiometrically driven thresholds, or shifts in limitation, across ecological levels of organization presents a fertile theoretical ground for further study.

## Case studies

We compiled and described several case studies to illustrate the potential transferability of TERs to a diversity of ecosystems and ecological and evolutionary processes (Table 1; Table S1). This compilation includes organismal examples ranging from microbes to trees and ecosystem functions from N-cycling to organic matter processing, in both terrestrial and aquatic habitats. Below, we expound on three examples from Table 1 to illustrate the TER concept at different levels of ecological organization and describe how TERs can influence micro- and macroevolutionary processes (Fig. 2).

### Lake eutrophication and cyanotoxin production

Absolute and relative concentrations of N and P have long been recognized for their role in lake productivity and eutrophication and therefore provide a useful starting place to translate the TER across levels of ecological organization. For instance, constraints on N supply likely limit phytoplankton growth in P-replete Canadian prairie lakes (Hayes et al. 2019). Similarly, increased diffuse watershed-based N inputs likely increase productivity in P-rich lakes in Northern Ireland (Bunting et al. 2007). Lakes in watersheds with greater human activity may accumulate P faster than N (Yan et al. 2016), leading eutrophic lakes to have a lower total N:P ratio than less nutrient-rich ecosystems (Zhou et al. 2022).

Shifts in lake stoichiometry may mediate the production of harmful cyanotoxins and represent an ecosystem-level TER with far-reaching management implications. Cyanotoxins, secondary metabolites produced by cyanobacteria, can affect water quality and threaten human and other organisms’ health (Lopez-Rodas et al. 2008; Buratti et al. 2017). The chemical structure of cyanotoxins can vary from N rich to C rich, with molar C:N ranging from about 2 to 43 (Waal et al. 2014). This variation in cyanotoxin structure sets up the potential for a TER. Specifically, the cyanobacteria *Microcystis* spp. produces more cyanotoxin microcystin under lower water column C:N (Waal et al. 2009; Osburn et al. 2022), and microcystin production can decrease nonlinearly with *Microcystis* spp. C:N and C:P tissue stoichiometry, suggesting a shift from C to N or P limitation may drive nonlinear cyanotoxin production. Other opportunities to translate TERs of cyanotoxin production exist beyond *Microcystis* spp. and fresh waters. For example, *Aphanizomenon* spp. (Wagner et al. 2023) and *Dolichospermum* spp. (Kramer et al. 2022) produce more cyanotoxins under N-rich environmental conditions. In contrast, C-rich cyanotoxins (e.g., karlotoxins and amnesic shellfish poisoning toxin) found in oceans generally increase under conditions of N or P limitation (Waal et al. 2014). These observations prompt multiple questions: do these genera support thresholds with respect to cyanotoxin production and relative nutrient availability? If so, what are the thresholds and how do they compare to *Microcystis* spp.? How does the chemical composition (i.e., N rich vs. C rich) of the cyanotoxin influence the shape of the response? Importantly, detecting a TER of cyanotoxin production based on lake N:P concentrations may be hampered by the N-fixing capabilities of many cyanobacteria, which increase cellular N concentrations independent of environmental N supply (Osburn et al. 2022). Nevertheless, understanding the relative availability of N and P has been instructive in predicting and managing harmful algal blooms. Thus, placing this understanding within a TER framework may provide a path forward by focusing on drivers of shifts in limitation of cyanotoxin production, whether these drivers are acting on environmental supply or species demand for energy and nutrients.

### Alder and salmon influences on nitrogen dynamics

In terrestrial ecosystems, plants that host N-fixing symbionts can play a large role in the relative availability of N to C or P (Vitousek et al. 2002). Alder (*Alnus* spp.) are shrubs that form a symbiotic relationship with N-fixing bacteria. Because this symbiosis results in abundant inorganic N in the soil near extensive patches of alder, streams in catchments with high percent cover of alder experience large amounts of N export from their surrounding watershed (Stottlemeyer and Toczydlowski 1999). Particularly in boreal and coastal rainforest headwater streams that lack marine-derived N from migratory salmon (Shaftel et al. 2012), alder may promote P limitation of aquatic ecosystem processes through N-saturation (Shaftel et al. 2012; Devotta et al. 2021), although nutrient limitation by N and P may also depend on algal community composition (Volk et al. 2008). Alder cover is also related to N fixation rates, with in-stream dissolved inorganic N linearly increasing with alder cover in the catchment and benthic N fixation rates linearly decreasing with increasing alder cover (Hiatt et al. 2017).

While increases in alder cover are associated with linear trends in some ecosystem-level responses among streams, shifts in alder cover and their resultant effects on stream ecosystem stoichiometry can create thresholds by generating nonlinear dynamics over time. Particularly under a warming climate, alder expansion could result in higher levels of N export from watersheds (Salmon et al. 2019), where the amount of alder cover in the watershed initiates a threshold response by increasing stream water N:P. This nonlinear response of stream water N:P to alder cover may, in turn, influence microbial metabolic activity via a shift from N to P limitation (Devotta et al. 2021). Alder cover can also initiate stoichiometrically driven thresholds by enhancing the growth of other terrestrial plant species. When the effects of salmon marine-derived N on white spruce were investigated at sites with and without alder, Helfield and Naiman (2002) found that foliar C:N in white spruce (*Picea glauca*) was highest at sites without alder or salmon present, and alder and salmon interacted to influence white spruce basal area growth.

### The Cambrian explosion

The end of the Proterozoic eon (~ 540 MYA) was marked by extensive erosion of terrestrial material into the ocean, causing influxes of calcium (Brennan et al. 2004), phosphate (Cook 1992; Brasier and Callow 2007), and other ions (iron, strontium, bicarbonate) into the oceans that exceeded any previous period of Earth’s history in the geological record (Squire et al. 2006). These nutrients triggered an explosion of algae and cyanobacteria (Squire et al. 2006; Canfield et al. 2007; Lyons et al. 2014) which produced a large increase in oxygen from photosynthesis (Campbell and Squire 2010). The combined effects of increased oxygen, calcium, and phosphate causing a shift in, or release from, elemental limitations may have been a driving mechanism for the Cambrian faunal “explosion” and the initiation of biomineralization (Elser et al. 2006). This explosion resulted in a threshold response of the largest diversification event of animals at the global scale.

Dramatic changes in calcium and P availability facilitated major transitions in the evolution of animals, the evolution of exoskeletons, and possibly the evolution of higher growth and metabolic rates of metazoans (Elser et al. 2006) and the evolution of self and non-self recognition (Fernàndez-Busquets 2010). Increased phosphate and calcium levels may have favored the evolution of phosphatized and calcified exoskeletons. Hydroxyapatite (Ca_10_(PO_4_)_6_(OH)_2_) is the primary constituent of vertebrate bones and detoxifies excess levels of P; its expanded use as a structural material may have facilitated increased diversification rates during this era (Cohen et al. 2017). Similarly, selection to reduce the toxicity of calcium ions to normal cell function may have caused the evolution of existing biochemical pathways to mineralize calcium. Shells composed of calcium carbonate (CaCO_3_) detoxify both calcium and oxygen ions, and supersaturation of CaCO_3_ permitted animals in all phyla to precipitate CaCO_3_ for the first time (Squire et al. 2006), consistent with the first appearance of skeletal marine phyla from ∼545 Ma (Martin et al. 2000). At the beginning of the Cambrian period, large increases in P, the most limited nutrient in the oceans over geological timescales, in concert with increased oxygen levels, would have permitted higher animal growth rates by relieving severe P limitation (Elser et al. 2006). Calcium increases the binding forces between calcium-dependent cell adhesion molecules; thus, large increases in calcium concentrations could have contributed to the Cambrian explosion by increasing adhesion between cells, thus facilitating the evolution of self and non-self recognition, and possibly multicellularity (Fernàndez-Busquets 2010).

Vast scale geological processes occurring at the end of the Phanerozoic permitted cascading and nested geochemical and biological feedback loops (e.g., evolutionary arms race, evolution of Hox genes) that together generated the Cambrian explosion (Smith and Harper 2013). Thus, stoichiometric constraints alone cannot explain this diversification event, but geological processes that released elements initiated the chain of events that led to the Cambrian explosion (Smith and Harper 2013), and elemental availability had fundamental impacts on the directions of further diversification. The stoichiometric drivers of the Cambrian explosion were unprecedented global increases in oxygen, calcium, and P, leading to the largest diversification event of animals. By any measure, the Cambrian explosion—an evolutionary event with fundamental and lasting impacts on the Earth’s biodiversity—represents a nonlinear increase in diversification rate influenced by stoichiometric shifts in resource limitation.

### Evolutionary thresholds

TERs may exist in an evolutionary context when organismal fitness or diversification varies with the stoichiometry of resources or the environment in a way that facilitates nonlinear evolutionary responses. At a microevolutionary level, components of fitness, such as somatic growth rate and fecundity, can vary with resource stoichiometry via the TER. The shape of growth or fecundity reaction norms over gradients in resource or environmental stoichiometry can therefore dictate whether a threshold on evolutionary responses will exist. For example, if an organism’s fitness does not vary with resource stoichiometry, it is unlikely that an evolutionary TER acts on that organism (Fig. 2a–d). Trinidadian guppies, for instance, vary in body stoichiometry among populations, but diet stoichiometry does not appear to have a large influence on growth rates or body stoichiometry (El-Sabaawi et al. 2012) (Fig. 2a–d).

If fitness is optimized at a single stoichiometric ratio with symmetric decreases in fitness away from this optimum TER, phenotypic or genotypic responses may be linear when sampled over a spatial gradient in stoichiometry (Fig. 2e–h). However, even if spatial patterns suggest a lack of a threshold, evolutionary rate may still vary with resource stoichiometry in a nonlinear fashion (Fig. 2e–h). One example where this relationship may occur is in the zooplankton *Daphnia pulicaria*, whose growth and fecundity are limited at both high and low C:P. Growth rates and fecundity in populations of *Daphnia* may vary in their TER over both time and space due to agricultural intensification promoting low resource C:P; however, the percentage of the population comprising homozygotes at a gene linked to P use efficiency, *Pgi*, to resource C:P appears to be linear (Moody et al. 2022) (Fig. 2e–h). Nonetheless, resurrection of resting eggs from dated sediment cores has revealed nonlinear dynamics in the rate of evolutionary change over time within the population of *Daphnia pulicaria* from a lake that became increasingly eutrophic over time (Frisch et al. 2014) (Fig. 2e–h).

Asymmetric reaction norms could lead to both nonlinear phenotypic responses over spatial gradients and evolutionary responses over time (Fig. 2i–l). Trees in the genus *Geissois*, which colonized the island of New Caledonia, radiated into 13 species from a single common ancestor. In New Caledonia, *Geissois* spp. encountered ultramafic soils with relatively high amounts of toxic metals like nickel (Ni) and low amounts of nutrients such as N, P, and K. Above the threshold for Ni toxicity, *Geissois* spp. populations evolved several distinct strategies for surviving on these soils, ultimately leading to an increase in speciation rate in these environments (Pillon et al. 2014) (Fig. 2 l). However, it is not possible to disentangle whether this nonlinear evolutionary response was solely driven by stoichiometry per se or the ecological opportunity associated with colonizing novel environmental characteristics. Links between micro- and macroevolutionary processes can be complex and discontinuous (Gould et al. 1998; Simons 2002). However, if influences on organismal fitness also have implications for speciation or extinction rates, they can influence macroevolutionary patterns (Rabosky and McCune 2010). The logic of the *Geissois* tree example follows this pattern—local adaptation to diverse stoichiometric conditions could accelerate speciation rates as well as influence adaptation within populations. Direct empirical evidence of stoichiometric shifts in resource limitation as a mechanism for evolutionary thresholds remains quite limited and represents a promising direction for future research.

## Literature review

We conducted a systematic literature review to evaluate whether translating the TER across levels of ecological organization could unify nutrient-focused ecological literature. Our literature review identified the extent to which ecological papers that focused on two essential elements, N and P, also considered the stoichiometry of these elements. We gathered papers using the search terms “nitrogen AND phosphorus” from the journals “Ecology Letters”, “Ecology”, “Functional Ecology”, and “Oikos” from the Web of Science in December 2022. We used these journals as a representative sampling of the ecological literature. The search generated 663 papers and included papers published from the years 1954–2022. We examined and read the titles and abstracts to assess suitability (i.e., “Was the paper an empirical study that focused on nutrients?”) with 563 considered appropriate. We then examined suitable studies more closely and answered the following questions: (1) Did the study measure a nutrient (e.g., N or P)? (2) Did the study measure both N and P? (3) Did the study report a stoichiometric ratio? (4) Was a stoichiometric ratio used in a statistical test? (5) Was a stoichiometric ratio used as (a) an explanatory variable, (b) a response variable, or (c) both? The database for the literature review can be accessed here: 10.5281/zenodo.14896078.

**Fig. 3:**
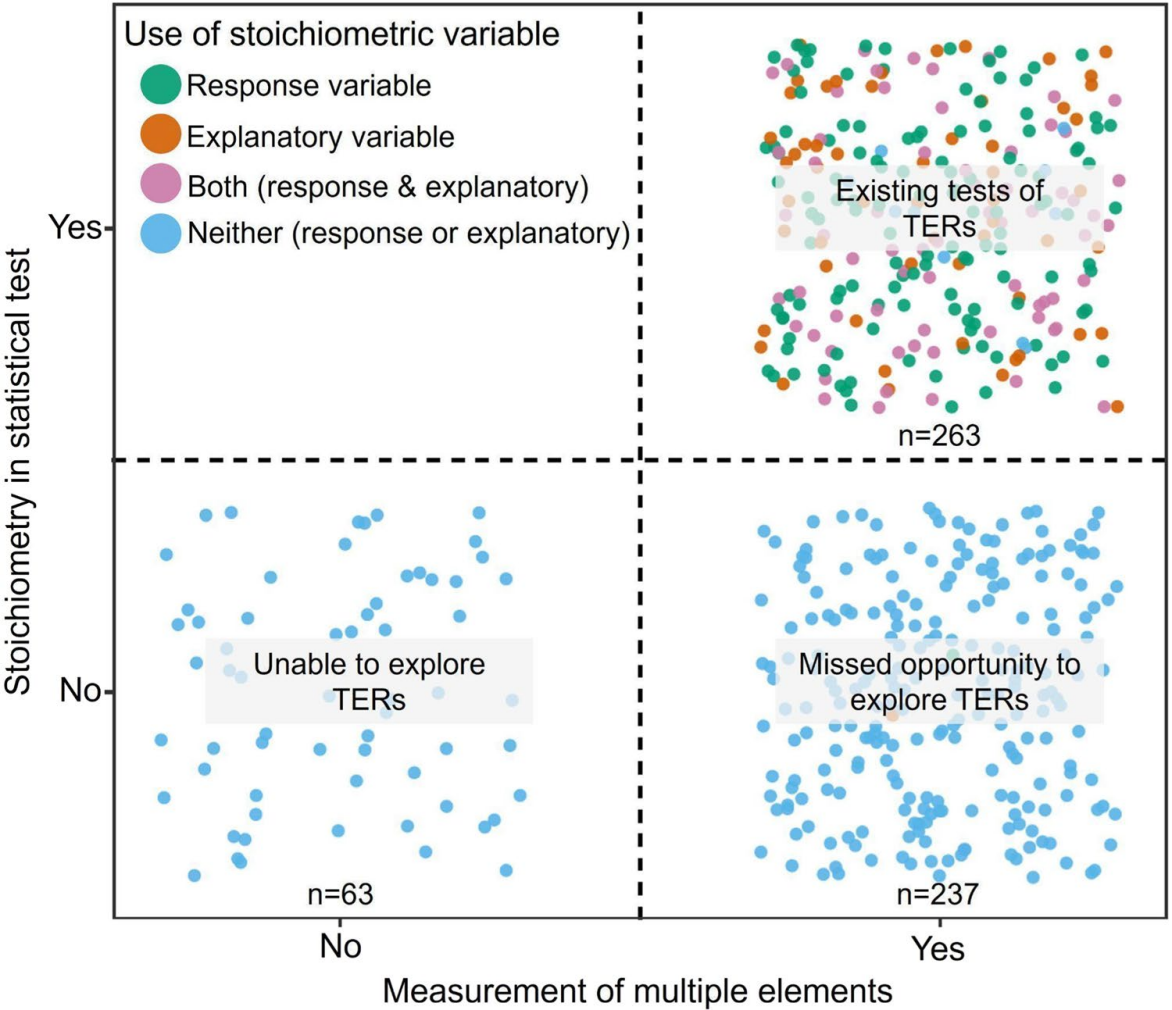
Ecological studies focused on nutrients and their potential to address threshold elemental ratio (TER) from the literature (*N* = 563). Studies are plotted based on whether multiple elements were measured and if elements were examined statistically as a ratio. Color refers to the use of variable within models. Text overlay provides interpretation of studies relevance to the TER concept based on placement within the plot

The findings from the literature analysis indicate that the TER concept can unify and be meaningfully applied to a considerable portion of existing nutrient-focused ecological literature and possibly catalyze future research on the topic. Specifically, we found that all studies included a topic relevant to nutrients in ecological systems. A small number of studies (*n* = 63, 11%) only examined a single nutrient, and therefore were unable to test stoichiometric questions (Fig. 3). A greater proportion of papers measured multiple elements, but did not present or analyze these elements as stoichiometric ratios (*n* = 237, 42%). Therefore, the studies within this category missed an opportunity to explore TERs despite possessing the necessary information to do so (Fig. 3). Nearly half of the studies (*n* = 263, 47%) explored TERs or could have explored TERs by using a stoichiometric ratio as an explanatory or response variable (Fig. 3). Although our consideration of the TER is focused on the stoichiometric ratio as the explanatory variable, we included both explanatory and response variables in this analysis as a more comprehensive estimation of studies that had the potential to investigate the concept. The findings of our literature search are promising for applying the extended TER concept. However, these findings only offer a glimpse into the ways one might examine and estimate TERs more broadly. Future work could compile the nutrient-focused ecological literature and perform a meta-analysis to quantify the occurrence of TERs and TER properties such as sensitivity and shape. Such a database could also be used to examine if and how TERs translate across levels of ecological organization.

## Modeling scaled threshold elemental ratios

**Fig. 4:**
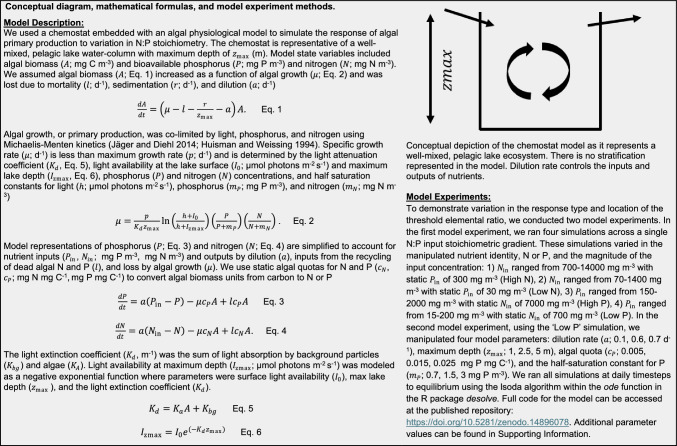
Conceptual diagram, ordinary differential equations, and outline of modeling experiments

Here, we provide an example analysis of an ecosystem-level TER using a lake ecosystem process model embedded with an algal physiological model. Using this process model, we evaluated the response of lake ecosystem gross primary productivity (GPP) to a gradient of supply N:P stoichiometry (see Fig. 4 for methodological details). Our goals with this model were to: (1) illustrate the presence of a TER for an ecosystem-level response; and (2) provide examples of mechanisms acting across levels of ecological organization that may drive variation in the shift in limitation underlying the TER. Specifically, we were interested in how the location and response type (i.e., *x* and *y* value) of the threshold may vary under different parameterizations using a well-established theory (Fig. 1; Klausmeier et al. 2008).

**Fig. 5:**
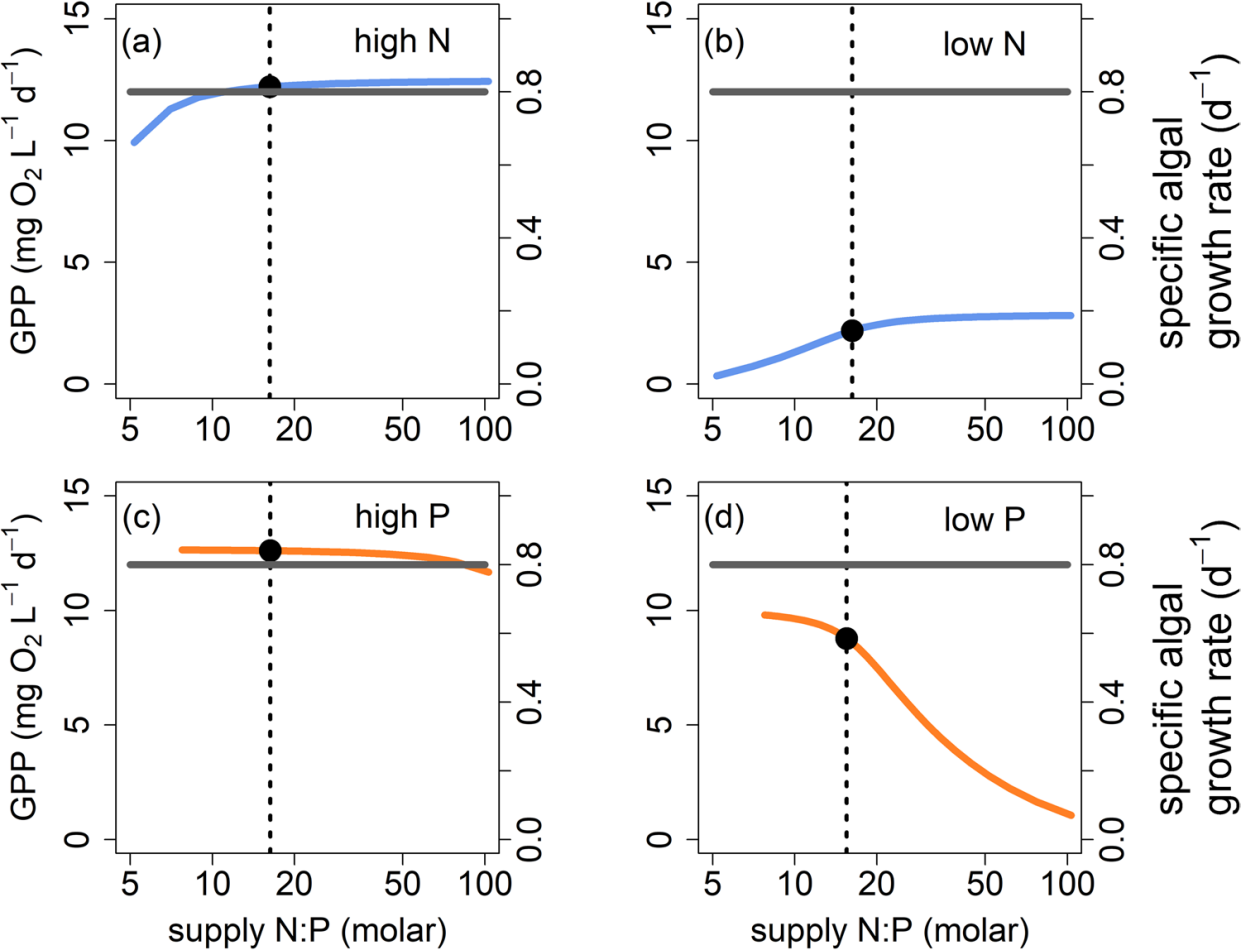
Results from the first model experiment to demonstrate how the identity and magnitude of the manipulated nutrient (N vs P) alters the response type and location of the threshold elemental ratio (TER) describing lake gross primary production (GPP; mg O2 L^−1^ d^−1^) across a supply N:P stoichiometric gradient. (**a)** N input ($N$_in_) ranged from 700 to 14000 mg m^−3^ with static P input ($P$_in_) of 300 mg m^−3^ (high N, solid blue line), (**b**) $N$_in_ ranged from 70 to 1400 mg m^−3^ with static $P$_in_ of 30 mg m^−3^ (low N, solid blue line), (**c**) $P$_in_ ranged from 150 to 2000 mg m^−3^ with static $N$_in_ of 7000 mg m^−3^ (high P, solid orange line), (**d**) $P$_in_ ranged from 15 to 200 mg m^−3^ with static $N$_in_ of 700 mg m^−3^ (low P, solid orange line). The dotted line in all panels marks the *x* value of the threshold. The solid gray line marks algal specific growth rate (d^−1^). A TER occurred at the ecosystem level (GPP response), but not at the organismal level (growth rate response). The solid point is the *x* and *y* value of the TER

**Fig. 6:**
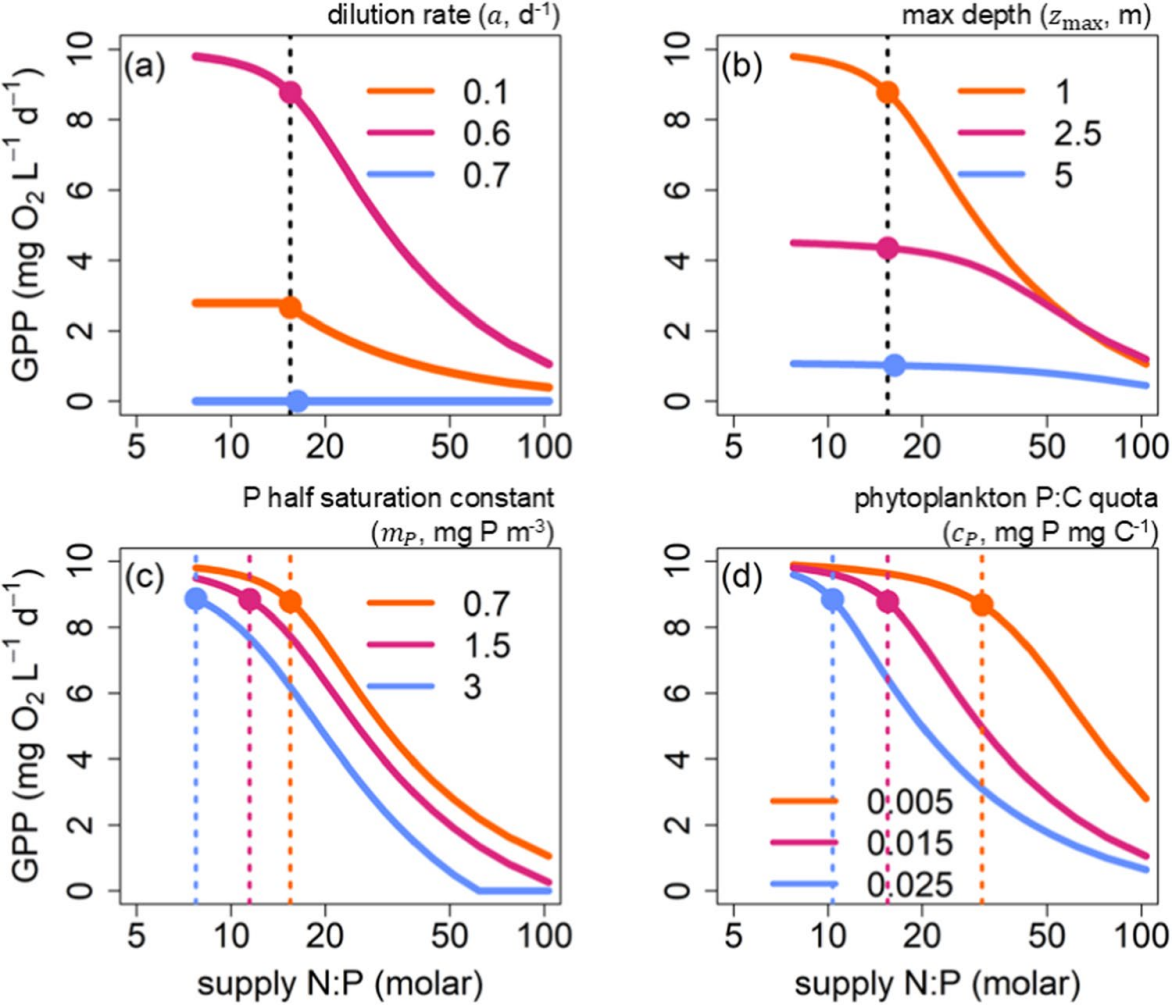
Using the “low P” simulation from the first model experiment, results from the second model experiment demonstrated how different mechanisms acting across ecological levels can alter the response type and shift the location of the TER. (**a)** Dilution rate ($a$; 0.1, 0.6, 0.7 d^−1^), (**b**) maximum depth ($z$_max_; 1, 2.5, 5 m), (**c**) the half-saturation constant for P (${m}_{P}$; 0.7, 1.5, 3 mg P m^−3^), (**d**) algal quota (${c}_{P}$; 0.005, 0.015, 0.025 mg P mg C^−1^). The dotted lines mark the *x* values of the TERs. The solid points are the *x* and *y* values of the TERs

Model simulations revealed that GPP responded nonlinearly to a gradient of supply N:P, indicating the presence of a TER driven by shifts in limitation between N and P (Fig. 5b, d, Fig. S1). However, the presence of nonlinearity and the type of nonlinear response was influenced by whether N or P was manipulated and the absolute concentration of the nutrient (Fig. 5). Gross primary production responded logistically across a stoichiometric gradient at low absolute concentrations of N and P (Fig. 5b, d). In these simulations, the TER (*x* value) was agnostic to both the identity and magnitude of the nutrient supplied and was always ~ 16 N:P. However, the GPP rate at which the threshold occurred (*y* value) did respond to nutrient identity and magnitude (Fig. 5b, d). In contrast, high absolute concentrations of N and P resulted in the absence of a nonlinear response of GPP to a stoichiometric supply gradient despite the presence of a shift from N to P limitation (Fig. 5a, c). This absence of nonlinearity is due to high N and P concentrations switching the primary limitation of GPP from nutrients to light via algal self-shading. These results are consistent with previous research (Downing and McCauley 1992; Bergstrom 2010); a stoichiometric ratio of 16 is customarily used as the threshold between N and P limitation for algal dynamics (Redfield 1958). In this example, the TER is dictated by the parameterization of the algal quota (mg P mg C^−1^) and can also be interpreted as algal nutrient use efficiency (biomass production per unit limiting nutrient; Fig. 5; Fig. 6c). However, system-specific nuances, such as community composition, often lead to deviations from this broader generalization of 16, and model parameterization of algal stoichiometric traits can be implemented to reflect this variation in algal quota (Fig. 6; Reynolds 1992; Klausmeier et al. 2004).

We also demonstrated how a threshold may occur at one ecological level, but not another. Algal specific growth rate, an organismal physiological response, did not demonstrate a nonlinear response type to nutrient identity or magnitude (Fig. 5a–d). In contrast, GPP, an ecosystem-level response, demonstrated a nonlinear response in two of the four simulations (Fig. 5a–d). This discrepancy across ecological levels of organization occurs because algal specific growth rate is dictated by dilution rate in the model (i.e., lake water residence time) rather than stoichiometry. When nutrients become increasingly scarce, individual algae quickly reduce the available nutrients to maintain their growth rate. This maintenance of growth rate causes less biomass to be accrued (i.e., a smaller number of individuals in the population or community). This result highlights the importance of properly identifying the process that is limited. Here, the organismal growth rate is not limited; rather, the ecosystem-level process of GPP is limited via building biomass with a steady growth rate (Reynolds 1992).

Using the low P simulation, we show that other mechanisms acting across ecological levels can influence the response type, sensitivity, and location of the TER via their direct or indirect effects on shifts in resource limitation (Fig. 6). Parameters acting on the supply of nutrients and light, such as dilution rate and maximum lake depth, did not change the TER (*x* value) but did change the response type and *y* value of the threshold (Fig. 6a, b). Parameters which act on algal nutrient demand (algal quota and P half-saturation constant) altered both the response type and TER (Fig. 6c, d). Algal physiological traits that dictate nutrient demand were the only parameters which altered the *x* value of the threshold or TER. In addition to the identity and magnitude of element(s) that alter the stoichiometric gradient (Fig. 5), care must be taken to identify and explore the supply and demand mechanisms influencing the location of the TER and the shape of its response.

Using well-established theory (Klausmeier et al. 2004; Kelly et al. 2018; Olson and Jones 2022), the lake supply N:P-GPP model provides a robust example for translating the TER across ecological levels, which could be expanded upon with other model formulations, organisms, and ecosystems. For example, a model structure with the Droop formulation of algal physiology, which allows for flexible algal stoichiometry, may be warranted to properly capture the TER dynamics (Supplementary Information; Droop 1968). Future work could consider alternative model structures and empirical experiments to test and compare the model predictions of static vs. flexible algal stoichiometry. A process model approach allows for the development of a priori hypotheses regarding the response type and location of a TER that can then be empirically tested. Additionally, such an approach can explore potential mechanisms behind the TER at various ecological levels. Here, a process model can help identify limiting elements other than P that control ecosystem structure and function (e.g., iron in the open ocean (Tagliabue et al. 2017), silica in coastal marine environments (Humborg et al. 2000)), and thus, the appropriate stoichiometric gradient required to observe such phenomena.

## Considerations for testing and inference from the TER across ecological levels

We recommend that future studies seeking to broaden the TER consider data requirements, modeling and transformation options, and scale. Rigorous statistical testing will be necessary to examine whether thresholds exist, including threshold analysis, breakpoint analysis, or formal model fit comparisons, see (Dodds et al. 2010; Spake et al. 2022). Recent work (Isles 2020) broadly recommends that stoichiometric ratios be log transformed to better represent nutrient ratio information to examine ecological stoichiometry theory. This point is especially pertinent when considering nonlinearity of stoichiometric relationships because by linearizing the response curve, logarithmic transformations may visually conceal a threshold, but underlying nonlinearity and the presence and location of a threshold still exist in the data. However, Isles (2020) argues that threshold analysis is robust to transformation because transformation does not affect the underlying rank order of the data. In Figs. 4, 5, we do not log-transform the x-axes (stoichiometric ratios), but we do present the stoichiometric ratios on logged axes as we did not want to lose the visual aspect of the threshold and ensure proper interpretation of the nonlinear response (Menge et al. 2018). We encourage researchers to consider study system, experimental design, and data characteristics. For example, attention should be given to transformations of ratio data in meta-analyses or other efforts that encompass especially large ranges in ratio data that are especially sensitive to bias. To that end, Menge et al. (2018) highlighted the serious issue of misinterpretation of log-transformed ecological data, and we also encourage researchers to heed their advice with respect to graphical representation and interpretation of statistical analysis.

Distinguishing between the presence vs. absence of a stoichiometric threshold will be central to applying the TER across ecological levels of organization. The absence of a threshold may be due to either a non-stoichiometric driver (e.g., light or temperature limitation), or the response is linear, suggesting either the absence of a threshold or that the stoichiometric gradient is not exhaustive enough to determine the threshold. Additionally, a testable TER must be derived from a continuous gradient of stoichiometry as opposed to comparisons above and below some threshold because gradients are necessary to test for nonlinearity (Kreyling et al. 2014). Such gradients may be difficult to achieve at relatively larger spatial and temporal scales (macroecology and macroevolution), but data synthesis and collaboration across disciplines have the potential to meet and work toward this need (Collins et al. 2017).

Studies examining TERs at varying ecological levels of organization will need to collect elemental data (which many are already doing; Table 1; Table S1) and distinguish between stoichiometric vs. non-stoichiometric drivers. Alongside mechanistic experiments, measuring multiple elements will help distinguish whether elemental ratios themselves govern patterns or instead respond to other factors. Similarly, studies will need to consider whether one element in the ratio vs. both or even multiple elements are ultimately responsible for thresholds. Such approaches would ideally be able to examine responses to altered elemental concentrations separately from altered elemental ratios (Kominoski et al. 2015).

There appears to have been greater empirical progress examining ecological compared to evolutionary TERs, highlighting an opportunity for innovative work regarding the influence of stoichiometric change in evolutionary processes Fig. 2 (El-Sabaawi et al. 2023). In a number of examples, changing resource stoichiometry appears to be associated with nonlinear shifts in evolutionary rate, but clear evidence supporting this hypothesis is lacking (Table 1; Fig. 2). This lack of support is common when attempting to link ecosystem-level processes to evolutionary changes (Fig. 2) and represents an exciting research frontier. In particular, experimental evolution studies paired with observational field evolutionary data could provide evidence to either support or refute the role of TERs in evolutionary processes. Selection of organisms which have relatively short generation times and a strong response to constraints in resource stoichiometry could yield evidence at the microevolutionary scale. At the macroevolutionary scale, phylogenetic analyses paired with empirical support for stoichiometry as the driver for speciation could provide evidence to support or refute TERs operating at this large scale.

## Conclusion

Collectively, we highlight how TERs may be pertinent across the ecological hierarchy and evolutionary processes. We also present evidence that such stoichiometrically driven thresholds can express counter-intuitive patterns when transferred across ecological levels, underscoring the need to better understand the mechanisms underpinning TERs and how such phenomena govern nonlinearity in ecology and evolution. Given the potential for applying TERs across ecological levels of organization, our group has provided recommendations for experimental and analytical approaches to encourage further development of and gain insight into the applicability of a broadened TER. Moving forward, we recommend that researchers examining stoichiometrically driven thresholds be thoughtful about which ecological levels they examine, spatial and temporal scale dependency, and how they consider the role of elemental ratios in ecological and evolutionary processes.

## Supplementary Information

Below is the link to the electronic supplementary material.

Supplementary file1 (DOCX 5043 KB)

## Data Availability

All data can be accessed at the published repository: 10.5281/zenodo.14896078.

## Code availability

Full code for the model can be accessed at the published repository: 10.5281/zenodo.14896078.
