# Supplementary material for: Nonlinear thinking in ecology and evolution: applying the threshold elemental ratio across levels of ecological organization
Source: Oecologia. 2025 Dec 8;208(1):10. doi: 10.1007/s00442-025-05842-w (PMC12686070; doi:10.1007/s00442-025-05842-w)
Supplement: Supplementary file 1 — Supplementary file1 (DOCX 5043 KB) [file 442_2025_5842_MOESM1_ESM.docx]

**Supplementary Information**

*Nonlinear thinking in ecology and evolution: Applying the threshold elemental ratio across levels of ecological organization*

Benjamin B. Tumolo*, Carly Olson*, Erin Larson, Halvor M. Halvorson, Catherine E. Wagner, Amy C. Krist, Felicia S. Osburn, Eric K. Moody, Linnea A. Rock, Uchechukwu Ogbenna, Eli Wess, Briante Najev, Anthony Pignatelli, Jessica Corman

**Model Experiment using Droop Formulation of Algal Physiology**

Another approach to modeling algal physiology is the Droop formulation (Droop 1968). The Droop formulation allows for flexible algal stoichiometry via additional state variables representing algal internal nitrogen (N) and phosphorus (P) concentration (i.e., cell quota). For an in-depth analysis of the application of the Droop formulation see Klausmeier et al. 2004a and Klausmeier et al. 2004b. Here, we replicated model experiment one from the main text using this flexible representation of algal stoichiometry and co-limitation between light, N, and P (Table S1):

$\frac{dA}{dt}=\left( \mu-l-\frac{r}{z_{\max}}-a \right)A$ Eq. 1

$\frac{dP}{dt}=a\left( P_{\mathrm{in}}-P \right)-V_{P}\left( \frac{P}{m_{QP}+P} \right)A+lAQ_{P}$ Eq. 2

$\frac{dN}{dt}=a\left( N_{\mathrm{in}}-N \right)-V_{N}\left( \frac{N}{m_{QN}+N} \right)A+lAQ_{N}$ Eq. 3

$\frac{dQ_{P}}{dt}=V_{P}\left( \frac{P}{m_{QP}+P} \right)-\mu Q_{P}$ Eq. 4

$\frac{dQ_{N}}{dt}=V_{N}\left( \frac{N}{m_{QN}+N} \right)-\mu Q_{N}$ Eq. 5

$u=\frac{p}{K_{d}z_{\max}}ln \left( \frac{h+I_{0}}{h+I_{\mathrm{zmax}}} \right)\left( 1-\frac{k_{QP}}{Q_{P}} \right)\left( 1-\frac{k_{QN}}{Q_{N}} \right)$ Eq. 6

$K_{d}={K_{a}A+K}_{bg}$ Eq. 7

$I_{\mathrm{zmax}}=I_{0}e^{(-K_{d}z_{\max})}$ Eq. 8

Briefly, we ran four simulations across a single N:P input stoichiometric gradient. These simulations varied in the manipulated nutrient identity, N or P, and the magnitude of the input concentration: 1) $N_{\mathrm{in}}$ ranged from 700-14000 mg m^-3^ with static $P_{\mathrm{in}}$ of 300 mg m^-3^ (High N), 2) $N_{\mathrm{in}}$ ranged from 70-1400 mg m^-3^ with static $P_{\mathrm{in}}$ of 30 mg m^-3^ (Low N), 3) $P_{\mathrm{in}}$ ranged from 150-2000 mg m^-3^ with static $N_{\mathrm{in}}$ of 7000 mg m^-3^ (High P), 4) $P_{\mathrm{in}}$ ranged from 15-200 mg m^-3^ with static $N_{\mathrm{in}}$ of 700 mg m^-3^ (Low P).

We found that patterns of gross primary productivity (GPP; mg O_2_ L^-1^ d^-1^) demonstrated by the Droop formulation were similar to those shown using static stoichiometry (Figure S2). In this exercise we used an ‘optimal N:P ratio’ (Klausmeier et al. 2004b) of ~21 and, intuitively, the TER shifted from 16 in the static model to this optimal N:P ratio of 21 (Figure S2). Parameterization choices regarding minimum or static quota will primarily determine the TER and these optimal ratios vary based on species (Klausmeier et al. 2004b). In both model structures the mechanism behind the TER is the same: a shift from N to P limitation (Figure S3). The shape of the TER in the Droop is similar to the static stoichiometry results. This is likely because we used a relatively low dilution rate ($a$=0.6), thus internal algal quota ($Q_{N}$: $Q_{P}$) followed that of supply N:P (Figure S4). In contrast, the y-value of the TER is generally shifted upwards; flexible stoichiometry allows for the internal quota to adjust to supply, nutrient use efficiency increases, and more carbon is fixed per unit N or P. A deep dive into GPP and consequences of different model structures on TERs is out of the scope of this paper. Nevertheless, this initial model comparison exercise highlights interesting similarities and differences between the two model structures that could be further examined with either a more thorough model exploration or experimental or observational data.

**Table S1**. Additional Examples of TERs.

Level of Org. = Level of biological organization (organismal, community, ecosystem); Study Grain Temporal = Temporal scale of individual observation units; Study Grain Spatial = Spatial scale of individual observation units; Study Extent Temporal = Temporal scale of entire study; Study Extent Spatial = Spatial scale of entire study; An. Scale = Analytical scale (Log = logarithmic, Add = additive); stoich = stoichiometry

| Topic | Elements | Ecosystem | Driver | Level of Org. | Grain | | Extent | | Response | Reference |
| --- | --- | --- | --- | --- | --- | --- | --- | --- | --- | --- |
|  |  |  |  |  | Temporal | Spatial | Temporal | Spatial |  |  |
| Predator introduction | C, P | Aquatic: temperate lake | Removal of planktivorous fish | **P**: Organismal (seston C:P)  **R**: Community (zooplankton biomass) | BACI design monthly to weekly | Fyke nets, Schindler traps | 4 years | 2 lakes | Threshold | Elser et al. 1998 |
| Trait evolution in *Daphnia* | C, P | Aquatic: temperate lake | Agricultural intensification | **P**: Ecosystem (shifting resource stoich via P addition)  **R**:Population (growht rate, P use efficiency) | Estimated year from Pb 210 dating of cores | 2-3 sediment cores from single lake | 1600 years | Single lake (South Center Lake, MN, USA) | Unknown | Frisch et al. 2014 |
| Watershed biogeochemistry | N, P | Aquatic: temperate rainforest streams | Alder cover in watershed | **P**: Organismal (Percent alder)  **R**: Ecosystem (Stream Nitrate:TP) | Seasonal | Watershed | 4 years | 26 streams over a 13,200 km^2^ landscape | Threshold | Devotta et al. 2021 |
| Mussels & nutrient recycling | N, P | Aquatic: rivers | Animals excreting at high N:P | **P**: Population (abundance) **R**: Ecosystem (nutrient availability) | 1 hr-18 days | 1500m2 | 18 days | 3 mid-size rivers | Threshold | Atkinson et al. 2013 |
| Cambrian explosion | C, P | Aquatic: spring-fed stream | Experimental manipulation of resource P | **P**: Ecosystem (P enrichment) **R**: Organismal (Microbialite C:P) | Snapshot at beginning and end of experiment | Single stromatolite from 20L container | 39 days | Recirculating mesocosm (32 20L containers) | Threshold | Elser et al. 2006 |
| Interaction between disease and food quality | C, P | Aquatic: lab experiment with *Daphnia* and *Pasteuria* sp. Bacterium | Resource C:P interaction with infection status | **P**: Organismal (Infection status by food C:P interaction) **R**: Organismal (mass, %N, %P) | 4 sampling days (days 4, 8, 15, 28) during the experiment | Individual organisms (10 individuals/replicate jar) | 28 days | Lab bottle assay with 3-5 replicates per experimental treatment combination | Threshold | Frost et al. 2008 |
| Test of Liebig's law of the minimum on mycorrhizal phenotypes | C, N | Terrestrial: grassland | Mycorrhizal plant tissue N:P as proxy for phenotype/limitation | **P**: Ecosystem (N and P availability)  **R**: Community (mycorrhyizal growth) | Single time point | Single rhizomes in individual plastic pots | Weeks to months (3 different experiments with different lengths) | 3 North American grasslands (Cedar Creek, Fermi, Konza) | Threshold | Johnson et al. 2015 |
| *Daphnia* life history and feeding behavior | C, P | Aquatic: ponds and lakes with laboratory reared organisms | Dietary C:P contents | **P**: Organismal (algal P content) **R**: Organismal (*Daphnia* life history) | Snapshot at beginning and end of experiment | Individual *Daphnia* | 6 days | 200mL containers in the lab | Threshold | Plath and Boersma 2001 |
| Body size influence on excretion stoich in neotropical fish and amphibians | N, P | Aquatic: 4th-order neotropical stream | Body size | **P**: Organismal (Body size) **R**: Organismal (N:P excretion ratio) | 3 sampling time points in the same year (January, February, March) | Individual organisms | 3 months | Stream reach | Linear increase of excretion rate N:P with body size | Vanni et al. 2002 |
| Microcystis growth rate and toxin production | C, N, P | Aquatic: monoculture bioassays | Resource N:P growth rate | **P**: Ecosystem (resource conc. and ratios of N and P) **R**: Population (growth rate, stoich, and toxin content | Every 2 days | NA | 12 days | NA | Threshold | Wagner et al., 2019 |

**Table S2.** Model parameters and values.

| **Symbol** | **Variable** | **Unit** | **Value** |
| --- | --- | --- | --- |
| *State Variables* |  |  |  |
| *A* | Algal biomass | mg C m^-3^ | -- |
| *P* | Dissolved inorganic phosphorus | mg P m^-3^ | -- |
| *N* | Dissolved inorganic nitrogen | mg N m^-3^ | -- |
| $Q_{P}$^ | Internal cellular P quota | mg P mg C^-1^ | -- |
| $Q_{N}$^ | Internal cellular N quota | mg N mg C^-1^ | -- |
| *Parameters* |  |  |  |
| $K_{a}$ | Light attenuation coefficient for algae | m^2^ g C^-1^ | 2.5*10^-4^ |
| $K_{bg}$ | Background light attenuation coefficient | m^-3^ | 0.38 |
| I_0_ | Light intensity at the surface | µmol photons m^-2^ s^-1^ | 400 |
| $z_{\max}$ | Lake depth | m | 1 |
| $p$ | Maximum production rate of algae | day^-1^ | 1 |
| $h$ | Half-saturation constant for light | µmol photons m^-2^ s^-1^ | 36 |
| $m_{P}$ | Half-saturation constant for P | mg P m^-3^ | 0.7 |
| $m_{N}$ | Half-saturation constant for N | mg N m^-3^ | 5 |
| $l$ | Loss rate of algae | day^-1^ | 0.1 |
| $r$ | Sinking rate of algae | day^-1^ | 0.1 |
| $a$ | Dilution rate | day^-1^ | 0.6 |
| $P_{\mathrm{in}}$ | Dissolved P concentration of supply | mg P m^-3^ | P High: 150-2000  P Low: 15-200  N high: 300  N low: 30 |
| $N_{\mathrm{in}}$ | Dissolved N concentration of supply | mg N m^-3^ | N High: 700-1400  N Low: 70-1400  P high: 7000  Plow: 700 |
| $c_{P}$ | Phosphorus to carbon algal quota | mg P mg C^-1^ | 0.015 |
| $c_{N}$ | Nitrogen to carbon algal quota | mg N mg C^-1^ | 0.1 |
| $k_{QP}^$ | Minimum P quota | mg P mg C^-1^ | 0.001 |
| $k_{QN}$^ | Minimum N quota | mg N mg C^-1^ | 0.009 |
| $V_{P}$^ | Maximum P uptake rate | mg P m^-3^ day^-1^ | 0.4 |
| $V_{N}$^ | Maximum N uptake rate | mg N m^-3^ day^-1^ | 1 |
| $m_{QP}$^ | Half-saturation constant for P | mg P m^-3^ | 5 |
| $m_{QP}$^ | Half-saturation constant for N | mg N m^-3^ | 30 |

^State variables and parameters unique to model version with Droop formulation


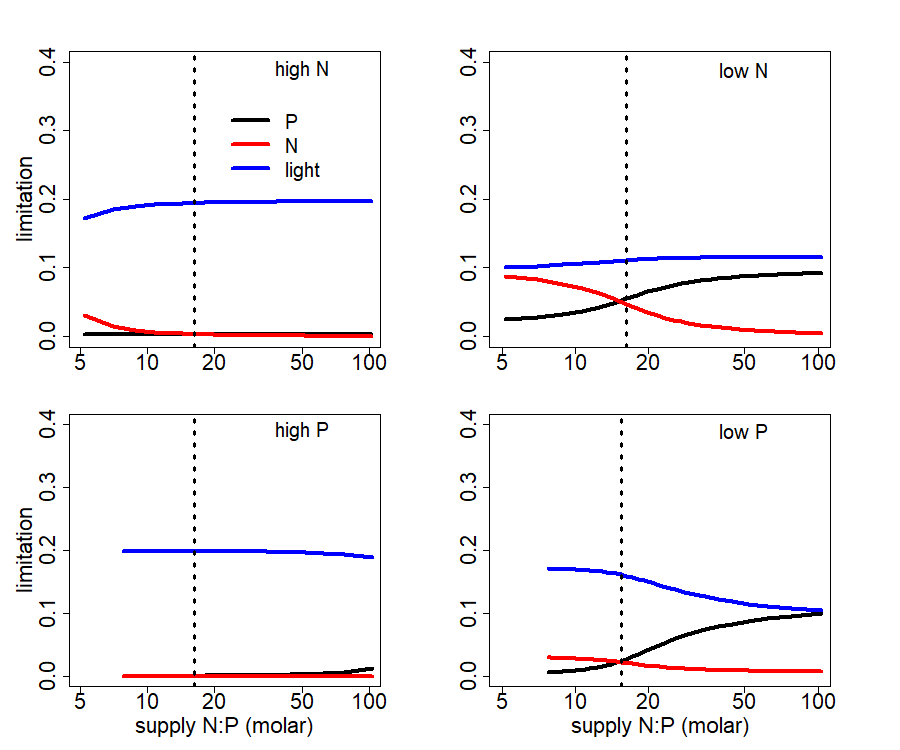


**B**

**A**

**D**

**C**

**Figure S1.** Limitation of algal gross primary productivity (GPP; mg O_2_ L^-1^ d^-1^) from the first model experiment demonstrating how the identity and magnitude of the manipulated nutrient, nitrogen (N) vs. phosphorus (P), alters the response type and location of the threshold elemental ratio (TER). Blue solid line = light limitation, black solid line = P limitation, and red solid line = N limitation. The black dotted line indicates the x-value of the TER and is where the shift from N to P limitation occurs. **A)** high N scenario, **B)** low N scenario, **C)** high P scenario, and **D)** low P scenario. The limitation value is relative and should only be compared within a panel, not between panels.

**B**

**A**


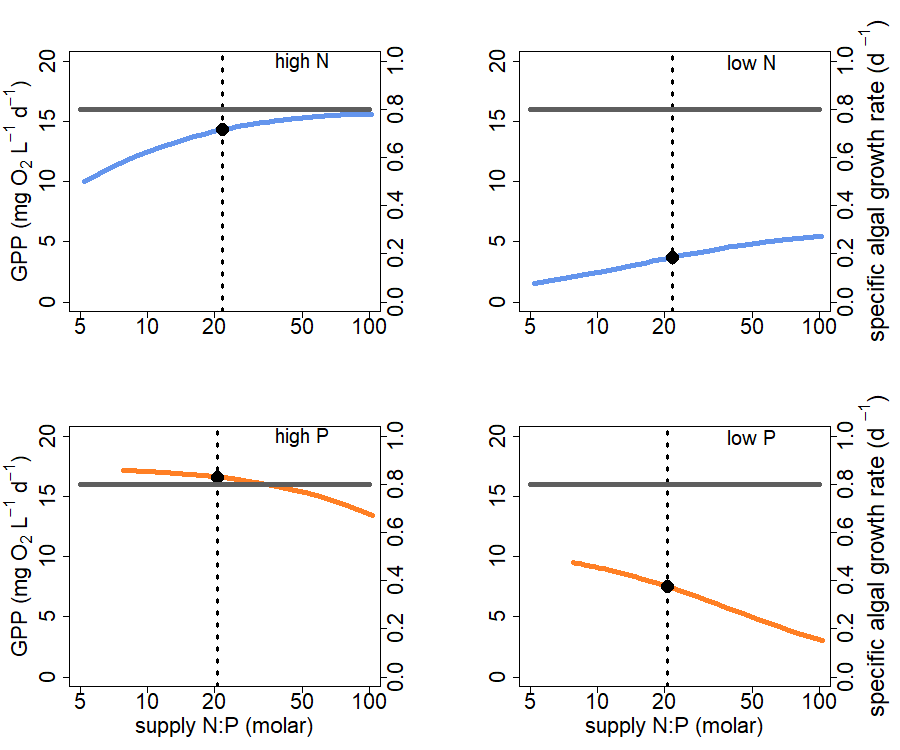


**D**

**C**

**Figure S2.** Response of gross primary productivity (GPP; mg O_2_ L^-1^ d^-^1) to a supply N:P stoichiometric gradient using the Droop formulation (i.e., flexible algal stoichiometry). **A)** $N_{\mathrm{in}}$ ranged from 700-14000 mg m^-3^ with static $P_{\mathrm{in}}$ of 300 mg m^-3^ (High N, solid blue line), **B)** $N_{\mathrm{in}}$ ranged from 70-1400 mg m^-3^ with static $P_{\mathrm{in}}$ of 30 mg m^-3^ (Low N, solid blue line), **C)** $P_{\mathrm{in}}$ ranged from 150-2000 mg m^-3^ with static $N_{\mathrm{in}}$ of 7000 mg m^-3^ (High P, solid orange line), **D)** $P_{\mathrm{in}}$ ranged from 15-200 mg m^-3^ with static $N_{\mathrm{in}}$ of 700 mg m^-3^ (Low P, solid orange line). The dotted line in all panels marks the x-value of the threshold. The solid gray line marks specific algal growth rate (d^-1^). A TER occurred at the ecosystem level (GPP response), but not at the organismal level (growth rate response). The solid point is the x- and y-value of the TER.


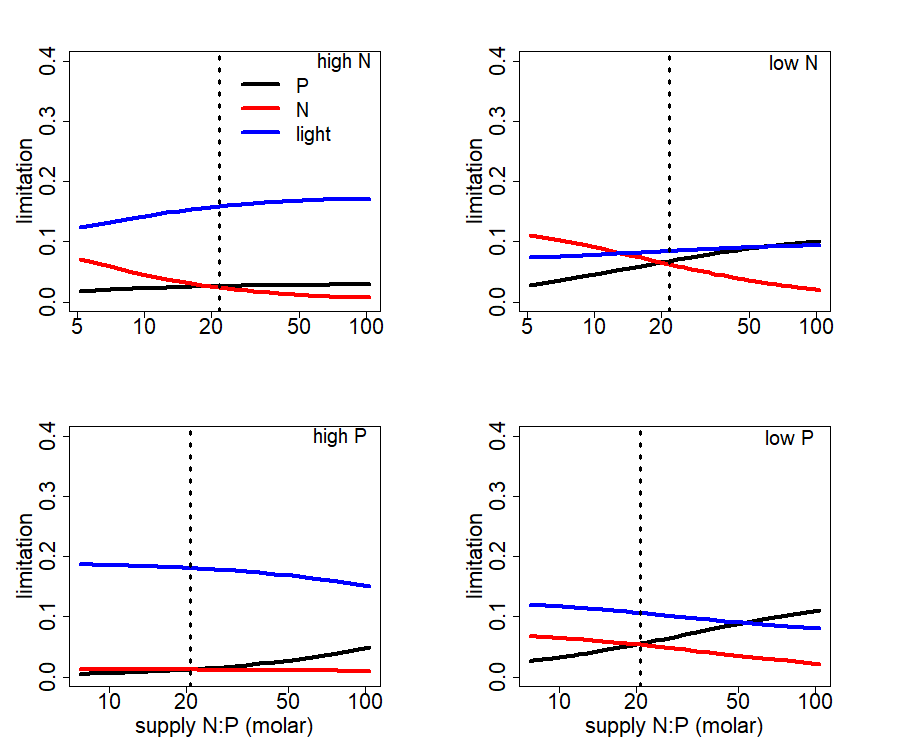


**C**

**D**

**B**

**A**

**Figure S3.** Limitation of gross primary productivity (GPP; mg O_2_ L^-1^ d^-^1) using the Droop formulation (i.e., flexible algal stoichiometry). Blue solid line = light limitation, black solid line = P limitation, and red solid line = N limitation. The black dotted line indicates the x-value of the TER and is where the shift from N to P limitation occurs. **A)** high N scenario, **B)** low N scenario, **C)** high P scenario, and **D)** low P scenario. The limitation value is relative and should only be compared within a panel, not between panels.


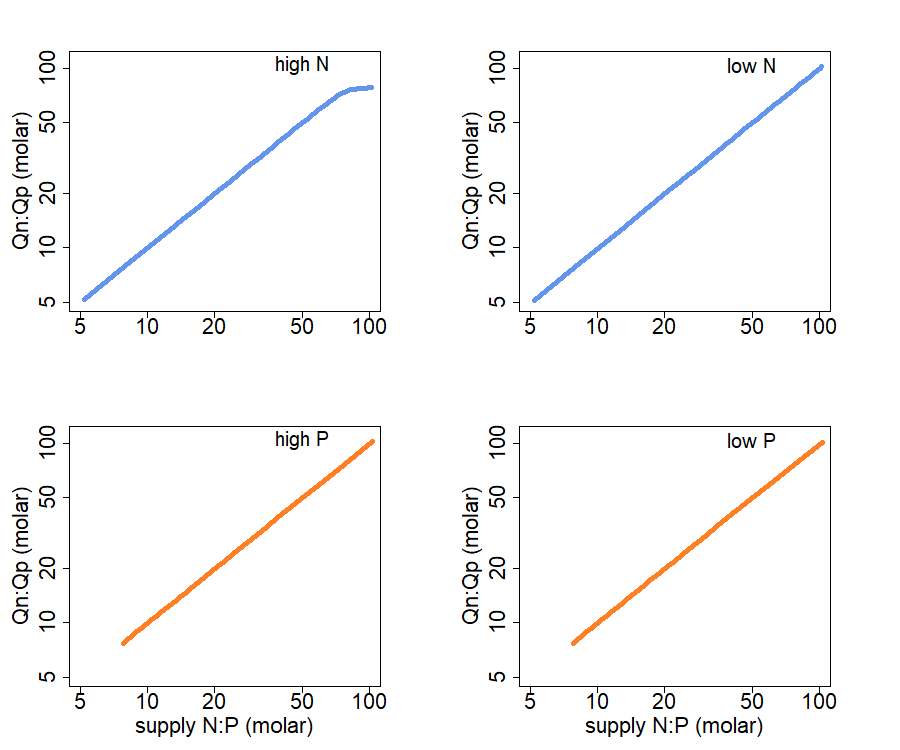


**D**

**C**

**B**

**A**

**Figure S4.** Algal quota ($Q_{N}$: $Q_{P}$) in response to a supply N:P stoichiometric gradient. Generally, there is a 1:1 relationship between algal quota and supply N:P. The pattern is due to the relatively low dilution (growth) rate $a$=0.6. A higher dilution rate would change this relationship. **A)** high N scenario, **B)** low N scenario, **C)** high P scenario, and **D)** low P scenario.

**Literature Cited**

Atkinson, C. L., Vaughn, C. C., Forshay, K. J., & Cooper, J. T. (2013). Aggregated filter‐feeding

consumers alter nutrient limitation: consequences for ecosystem and community dynamics. *Ecology*, *94*(6), 1359-1369.

Devotta, D. A., Fraterrigo, J. M., Walsh, P. B., Lowe, S., Sewell, D. K., Schindler, D. E., & Hu,F. S.

(2021). Watershed Alnus cover alters N: P stoichiometry and intensifies P limitation in subarctic streams. *Biogeochemistry*, *153*(2), 155-176.

Droop, M.R. (1968). Vitamin B12 and marine ecology. IV. The kinetics of uptake, growth, and

inhibition in *Monochrysis lutheri*. *Journal of the Marine Biological Association of the*

*United Kingdom.* 48:689-733.

Elser, J. J., Chrzanowski, T. H., Sterner, R. W., & Mills, K. H. (1998). Stoichiometric constraints

on food-web dynamics: a whole-lake experiment on the Canadian Shield. *Ecosystems*, *1*, 120-136.

Elser, J. J., Watts, J., Schampel, J. H., & Farmer, J. (2006). Early Cambrian food webs on a

trophic knife‐edge? A hypothesis and preliminary data from a modern stromatolite‐based ecosystem. *Ecology Letters*, *9*(3), 295-303.

Frisch, D., Morton, P. K., Chowdhury, P. R., Culver, B. W., Colbourne, J. K., Weider, L. J., &

Jeyasingh, P. D. (2014). A millennial‐scale chronicle of evolutionary responses to cultural eutrophication in Daphnia. *Ecology letters*, *17*(3), 360-368.

Frost, P. C., Ebert, D., & Smith, V. H. (2008). Bacterial infection changes the elemental

composition of Daphnia magna. *Journal of Animal Ecology*, *77*(6), 1265-1272.

Johnson, N. C., Wilson, G. W., Wilson, J. A., Miller, R. M., & Bowker, M. A. (2015).

Mycorrhizal phenotypes and the l aw of the minimum. *New Phytologist*, *205*(4), 1473-1484.

Klausmeier, C.A., E. Litchman, & S.A. Levin. (2004a). Phytoplankton growth and stoichiometry

under multiple nutrient limitation. *Limnology and Oceanography.* 49:1463-1470

Klausmeier, C.A., E. Litchman, T. Daufresne, & S.A. Levin. (2004b). Optimal

nitrogen-to-phosphorus stoichiometry of phytoplankton. *Nature.* 429:171-174.

Plath, K., & Boersma, M. (2001). Mineral limitation of zooplankton: stoichiometric constraints

and optimal foraging. *Ecology*, *82*(5), 1260-1269.

Vanni, M. J., Flecker, A. S., Hood, J. M., & Headworth, J. L. (2002). Stoichiometry of nutrient

recycling by vertebrates in a tropical stream: linking species identity and ecosystem processes. *Ecology Letters*, *5*(2), 285-293.

Wagner, N. D., Osburn, F. S., Wang, J., Taylor, R. B., Boedecker, A. R., Chambliss, C. K.,

Brooks, B. W., & Scott, J. T. (2019). Biological stoichiometry regulates toxin production in Microcystis aeruginosa (UTEX 2385). *Toxins*, *11*(10), 601.
